# ‘Collective making’ as knowledge mobilisation: the contribution of participatory design in the co-creation of knowledge in healthcare

**DOI:** 10.1186/s12913-018-3397-y

**Published:** 2018-07-25

**Authors:** Joe Langley, Daniel Wolstenholme, Jo Cooke

**Affiliations:** 1 Lab4Living, Art & Design Research Centre, Sheffield Hallam University, Sheffield, UK; 2 NIHR Devices for Dignity HTC, Sheffield Teaching Hospitals NHS FT, Sheffield, UK; 3 NIHR CLAHRC YH, Sheffield Teaching Hospitals NHS FT, Sheffield, UK; 4 Centre for Health and Social Care Research, Sheffield Hallam University, Sheffield, UK

**Keywords:** Coproduction, Co-creation, Participatory design, Empowerment, Co-design, Boundary object

## Abstract

The discourse in healthcare Knowledge Mobilisation (KMb) literature has shifted from simple, linear models of research knowledge production and action to more iterative and complex models. These aim to blend multiple stakeholders’ knowledge with research knowledge to address the research-practice gap. It has been suggested there is no ‘magic bullet’, but that a promising approach to take is knowledge co-creation in healthcare, particularly if a number of principles are applied. These include systems thinking, positioning research as a creative enterprise with human experience at its core, and paying attention to process within the partnership. This discussion paper builds on this proposition and extends it beyond knowledge co-creation to co-designing evidenced based interventions and implementing them. Within a co-design model, we offer a specific approach to share, mobilise and activate knowledge, that we have termed ‘collective making’. We draw on KMb, design, wider literature, and our experiences to describe how this framework supports and extends the principles of co-creation offered by Geenhalgh et al. [1] in the context of the state of the art of knowledge mobilisation. We describe how collective making creates the right ‘conditions’ for knowledge to be mobilised particularly addressing issues relating to stakeholder relationships, helps to discover, share and blend different forms of knowledge from different stakeholders, and puts this blended knowledge to practical use allowing stakeholders to learn about the practical implications of knowledge use and to collectively create actionable products. We suggest this collective making has three domains of influence: on the participants; on the knowledge discovered and shared; and on the mobilisation or activation of this knowledge.

## Background

The discourse in healthcare Knowledge Mobilisation (KMb) literature has grown from simple, linear models of research knowledge production and action, to more complex and iterative models supporting co-productive approaches [2]. These more complex models are described as Mode 2 learning where knowledge is created within the context of its use [1, 2]; working with those who are likely to use it [3, 4], and boundaries between knowledge producer and knowledge user are purposely blurred and utilised [5]. We define KMb as the activation of available knowledge within a given context. Within this are notions of recognition, movement, active use and context specificity of knowledge [6]. Equally there is an appreciation that KMb occurs on a variety of levels; personal, team and organisational but, as a social activity, is much more likely to happen via ‘bottom-up’ models, implying a growth or flow from personal upwards in scale.

We use the definition of Mode 2 (KMb) from Michael Gibbons who first put forward this description [7, 8]. In Mode 2, *‘…knowledge is produced in a context of application involving a much broader range of perspectives. It is transdisciplinary, not only drawing on disciplinary contributions but can set up new frameworks beyond them; it is characterised by heterogeneity of skills, by a preference for flatter hierarchies and organisational structures which are transient. It is more socially accountable and reflexive than Mode 1…’* .

Gibbons suggests that Mode 2 utilises a peer review system within the specific knowledge production community whilst also engaging a wider set of practitioners and experts, giving it an expanded system of quality control.

Such approaches aim to blend a variety of forms of knowledge from multiple stakeholders along with research knowledge to address the research-practice gap. However, because of the diversity of participants there is potential for misunderstanding and conflict [9], so the need to pay attention to how co-production is undertaken is of paramount importance in order to produce positive outcomes on service users, services and practice [3].

The wider field of Knowledge Translation has created much debate resulting in a spectrum from positivist, linear implementation models (Mode 1) to complex, social constructed, context sensitive and person centred knowledge mobilisation models (Mode 2). These varied schools of thought have resulted in a crowded landscape with over 60 models of implementation and KMb [10]. In a move towards consolidation, in the implementation science field (the study of KMb), Damschroder et al. [11] combined the pre-existing healthcare implementation models into an integrated framework that is highly complex consisting of 5 domains and 37 constructs. This framework identifies challenges in undertaking implementation in the real world that include contrasts in culture, trust, power, language and priorities between stakeholder groups. Another challenge is that knowledge has a tendency to stay in silos rather than being made visible and actively blended between groups, and there is often a mismatch between the end user’s understanding of research and researchers’ understanding of the policy and practice context [10, 11].

The scale of this consolidated framework recognises the complexity, but offers limited insight into how it can be operationalised to address the research-practice gap [11] and is located towards the implementation end of the spectrum described above. It has been suggested that there is no ‘magic bullet’, and that several approaches may be useful [12]. An approach recently described by Greenhalgh et al. [1] suggests that the best way to achieve impact and address the research-practice gap is to adopt a knowledge co-creation approach drawing and developing on existing principles of co-production. This paper clearly supports the development of mode 2 knowledge through a co-creation process which they define as ‘the collaborative generation of knowledge by academics working alongside stakeholders from other sectors’ (p 393). They suggest this approach moves beyond the notion of academics sitting in distant ‘ivory towers’ to one where dynamic and adaptive community-academic partnerships are nurtured and developed. They place emphasis on process, and suggest that co-creation is only likely to be successful if it adopts certain principles. These principles include:

- using a systems perspective that recognises the interrelationship between different parts of a system rather than focusing on any one part,
- positioning research as a creative enterprise that has human experience at its core, and
- paying attention to the quality of relationships within the partnership, applying facilitation techniques that consider power-sharing and utilise conflict as a positive force.

The Greenhalgh paper uses co-design examples to illustrate these points and arrive at these key principles, implying that, co-design approaches (across a range of disciplines) embody these principles.

**Fig. 1:**
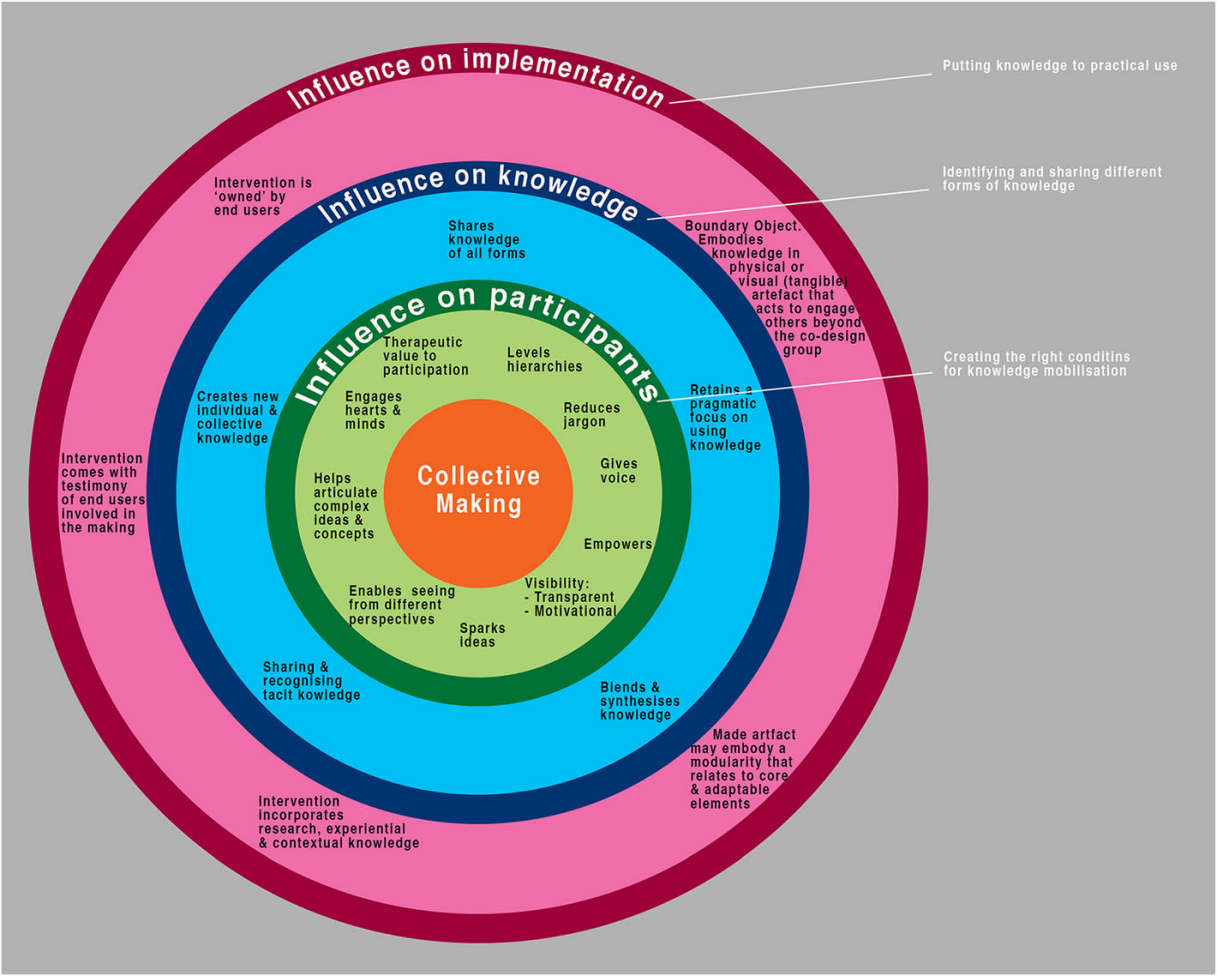
diagram illustrating the different domains of influence of collective making from a knowledge mobilisation perspective

Our discussion paper builds on this conclusion, and offers a more detailed framework of using ‘collective making’ as a specific co-design approach that we believe, as part of a creative process, addresses these principles. We have illustrated this framework below in Fig. 1.

This model is drawn and synthesised from the KMb and design literature as well as our own experiences. We suggest that, collective making influences knowledge mobilisation through a series of interactions at three levels.

The model of collective making has at its core the practice of collective making. It is this practice that has a direct influence on stakeholders themselves as co-creators in the process, creating the conditions for knowledge mobilisation.

This practice also exerts influence over the knowledge itself, allowing different forms of knowledge to be made visible, expressed, blended and activated or used, enabling stakeholders to learn practical implications of use.

The combination of the positive influences on participants and knowledge allows the third domain (the positive influence on implementation) to be realised, as it creates products, actionable tools and objects, in context, that contribute to the likelihood of uptake and use.

We do not intend to define all the underpinning theories and concepts that support this model. This model has emerged from reflection on practice, intradisciplinary discussion and a broad range of influences. We present it here for discussion, with some literature that explores possible candidate theories, as an opportunity to position collective making in the KMb literature and provoke a wider academic dialogue and feedback in response to this article.

We will start by defining the key terms of Design, Co-design, and Collective Making.

## Main text

### What is design?

‘Design’ is both a practice and a process. As a practice, it is something everybody can do yet is also a professional practice where those with training and extensive application gain considerable knowledge, skills and experience. As a process, design is an approach to problem solving that is human centred and collaborative. This should not be confused with participatory or co-design. The design process can (and often is) human centred and collaborative without involving the end user(s). Many professional designers use ergonomic data, computer simulations and other data, collaborating with those who commission and pay for their work, before direct consultation with end users.

Design helps to make ideas tangible [13], to develop practical and attractive propositions to users and customers [14] that are affordable and sustainable [15]. ‘Designerly strategies’ have been described by Stolterman and others [16–20] as being particularly suited to complex, ill-defined or *wicked* problems [21, 22]. Rittel [23] links design thinking and wicked problems, and describes wicked problems as ill defined, involving stakeholders with different perspectives, and having no ‘right’ or ‘optimal solution’ [24]. These attributes would appear to resonate in the context of healthcare settings, and there is increasing use of design and co-design in healthcare over the last 10 years [25].

This ability to deal with wicked problems stems from a solutions focused approach. As Lawson notes, with architects being a proxy for designers in this instance [26],:

*“…while the scientists focused their attention on discovering the rules, the architects were obsessed with achieving the desired result. The scientists adopted a generally problem focused strategy and the architects a solution focused strategy…”(p.23).*

A key component, and often cited as a defining characteristic, of design practice is prototyping.

### Prototyping

Prototyping exists as a spectrum of activities that cuts across a range from spontaneous to carefully planned, individual to collective. Brown [15] suggests:

*“The goal of prototyping isn’t to finish. It is to learn about the strengths and weaknesses of the idea and to identify new directions that further prototypes might take.”(p3).*

And that prototypes:

*“…command only as much time, effort, and investment as is necessary to* ***generate useful feedback*** *and* ***drive an idea forward*** *. The greater the complexity and expense, the more ‘finished’ it is likely to seem and the less likely its creators will be to profit from constructive feedback–or even to listen to it.”(p3).*

**Fig. 2:**
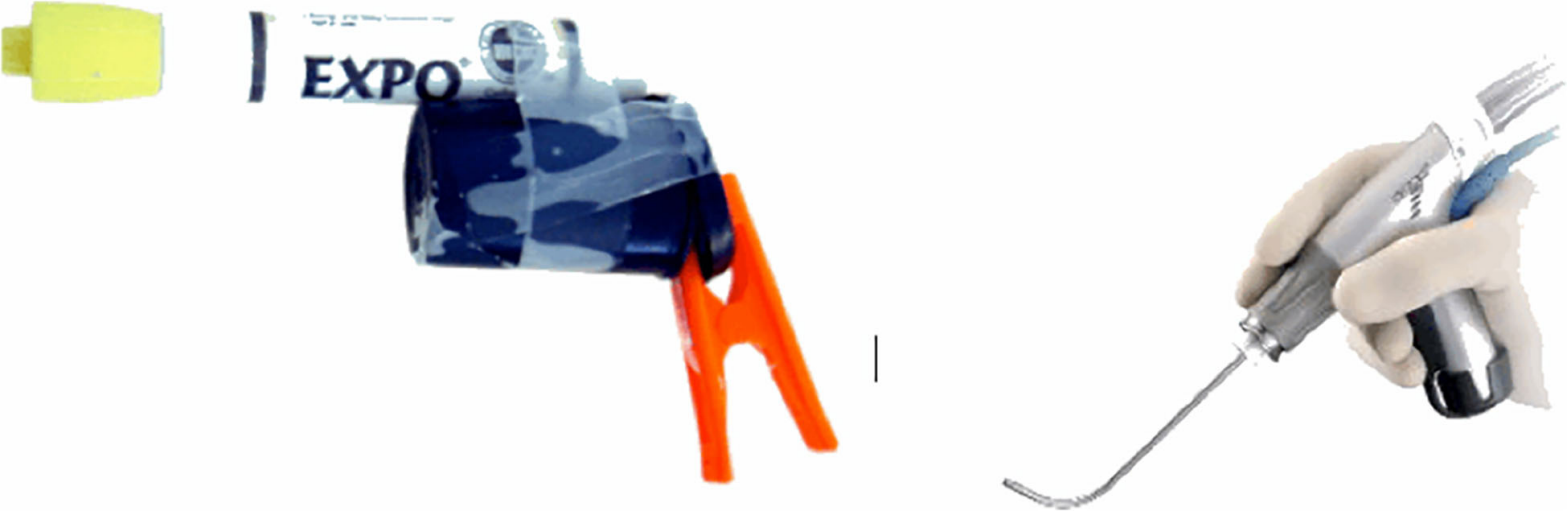
Early prototype (Left) and final product (Right) in a product development process by IDEO for Gyrus ENT Diego

Figure 2 illustrates a design mock-up or prototype used in the very early stages of idea development for a new product. It was quick to make as designers [15]were limited to the materials available and immediately conveyed a number of design ideas and limitations, such as physical size constraints if the device was to be a single-handed tool.

Prototypes are not always 3 dimensional (3D), nor are they merely an approach to testing design ideas or the functional limitations of physical or digital parameters. Prototypes and the process of making prototypes are fundamental to the way that designers think and communicate. For a designer, the process of drawing or making something is not to transcribe ideas from their heads but as a means of orchestrating a conversation with themselves (and, in co-design initiatives, with others). Externalising those emergent thoughts, making them tangible, allows designers to tap into their sensory (as well as cognitive) systems. This extends their thinking, distributing it between conception and perception, engaging both simultaneously and iteratively [27].

The designer moves through a series of drawings or prototypes asking “what if?”, and “would this work?”, each move creating a new problem to be described and solved and spinning out a web of consequences, implications, appreciations and further moves. Each move is a local experiment contributing to a wider global experiment of understanding about a bigger problem. The designer reflects on unexpected consequences or implications and responds to the hand, eye, brain dialogue [28]. From a cognitive science perspective, cognition is not a purely rational, ‘intra-mental’ activity but a practical, interactive activity. The mind working on its own is only a part of the full cognitive system [29]. The full system comprises a combination of thinking and action within a physical environment and in this way relates to Kolb’s or Dewy’s experiential learning cycle [30].

As a practice, design is essentially a translational discipline. It combines knowledge creation *and* knowledge use. It encompasses methods, knowledge and understanding from physical and social sciences, arts and humanities but always with a focus on delivering a solution that ‘works’ in practice.

An example of prototyping supporting KMb is to be found in the Head-Up project [31] in which a neck support was co-designed with patients who had Motor Neurone Disease (MND). This project included a series of co-design workshops in which people with MND and MND specialist health care professionals and researchers made things and prototypes to share their experiences, knowledge and ideas about their requirements related to neck support. Between workshops, designers made drawings and models that developed and/or challenged their ideas and comments. These design prompts tested the limits of the stakeholders’ imagination and assumptions. For example, one patient participant asked “What if I couldn’t nod but could shake my head from side to side?”. The designers made a prototype (the ‘R2D2’ model) for participants to try out and asked would it work for them? Their reaction was negative and so other ideas were pursued to a more successful conclusion. The designers response to the participants idea, making it tangible and visible, demonstrated that their suggestion were valued, listend to *and* acted upon. Without this, there might have always been the nagging doubt that an idea had been ignored and this would have undermined their participation in the shared process of knowledge mobilisation.

### What is co-design?

An important shift in design thinking occurred in the 1980s [32, 33]. There was a move away from the opinion of ‘users’ as passive recipients of design work (designing *for* people) to active participation in design processes (designing *with* people), and participatory design, or co-design, was developed [34]. We use the term participatory design here deliberately to link to the rich literature from this branch of design, but recognise, and intentionally use co-design in the rest of the paper as it has more traction in health. The two terms, we feel for a predominantly health audience, can be used interchangeably. This positions design practice in the co-creation arena, and aligns with the UK’s National Institute for Health Research (NIHR) policy about Patient and Public Involvement (PPI) practices and empowerment and emancipation [35]. Tsoukas and Vladimirou [36] define knowledge as *“… the individual ability to draw distinctions within a collective domain of action, based on an appreciation of context or theory or both…” (p979).* As such, co-design approaches align to critical theory and critical realism paradigms in the KMb landscape where methods are developed as a contribution to action and emancipation [37] with a particular emphasis on working with end users in solution focussed processes.

Co-design has an emphasis on process, where facilitation brings different participants together to elicit and share first-hand experiences and first-hand knowledge perspectives [38]. Co-design has therefore an ethos of empowerment and real engagement, placing such practices on the higher rungs of the ladder of participation described by Arnstein [39].

The co-design process recognises that the knowledge that stakeholders bring, is both explicit and tacit. Tsoukas and Vladimirous [36] describe this as ‘contextual appreciation’. Surfacing this knowledge and recognising its value are two key reasons for first-hand participation; it is often only participants who have mutual contextual appreciation, albeit from different perspectives, that recognise and value the tacit knowledge exposed when experiences are shared. This shared understanding of others experience is key in the early stages of building trust between diverse stakeholders and helps banish myths that constrain contextually sensitive solutions being developed.

Clearly knowledge and understanding are important in achieving a solution. However, where there are gaps in both, design takes a pragmatic and abductive approach – a range of prototypes are developed to see whether a design works and what it tells us about the gaps, as described in Lawson’s example of the architects. In doing so, design creates new knowledge that is visible to all participants, a visibility that is sustained through the ongoing physical presence of the prototypes.

### Collective making: A specific approach to co-design

In the context of healthcare, co-design is not new. A co-design initiative that has demonstrated a wide degree of use in healthcare is the Experienced Based Co-Design methodology (EBCD) [40, 41]. The EBCD approach was developed and in now availble as an online toolkit of replicable methods [42]. Similar to the business use of Design Thinking, the EBCD method and subsequent toolkits developed to share the methods of design and design processes without the costly support of professionally trained designers [43]. The process and use of EBCD is not always straightforward some projects have had limited tangible service improvement, others recognised the lack of ideation tools [44] and it is often described as ‘design like’ rather than designerly [45]. In EBCD activities, design methods have been distilled down into a simplified process to allow non-designers to use them but this removes a designer’s skills and experience from the process [46].

In a co-design process led by designers, designers employ a portfolio of techniques called generative methods [47]. These cover a wide range of activities through which co-design participants are ‘led’ that capture experiences, knowledge (explicit, tacit, embodied), habits, behaviours and ideas. The distinguishing feature of these designer facilitiated activities is that they involve some form of ‘making’. The making is used to help co-design participants explore, reflect and consider experiences, share, articulate and express them, and see how they compare and contrast with the experiences and perspectives of others. In this way, we enable the participant to think in a similar way to that of the designer. This is achieved in the number of iterative phases. The use of making stems from the assumption that the people in the process hold the relevant knowledge but are not explicit sources of information; they are limited in the ways of expression and communication, and many experiences and knowledge are tacit, embedded in the everyday. Designers facilitating such a co-design process will ask the participants a question or series of questions, asking them to make something to represent their response. We are not expecting them to transcribe a pre-existing answer but to begin to externalise their thoughts about the question, to use the making as an opportunity to reflect and to initiate a conversation with themselves.

Collective making is preceded by ‘skills building’ that enables confidence in using the media and approach within the individuals. It then ‘builds’ from the individual to the collective making each participant’s contribution visible. During the process, the focus is not on artistic qualities, but on what is being communicated. It is the combination of the made ‘things’ and the supporting description the maker gives the ‘thing’ they have made that is important. What is shared or learned is incorporated into subsequent rounds of making, where individual models are combined and blended into a negotiated model that embodies inclusion and a shared understanding whilst adhering to the meaning attributed by the original maker.

### So how does collective making relate to knowledge mobilisation?

The framework has, at its core, the notion of collective making which creates the right ‘conditions’ to ‘surface’ the knowledge within the participants, and influences what knowledge is shared, used, and applied. This ‘creative hermeneutics’ is based on the notion of ‘making is thinking’ [18], and that collective making co-creates knowledge and outputs. The framework suggests that collective making has three concentric domains of influence: on participants, because of the effect the creative practice has on them; on the knowledge it uncovers and creates, because it pragmatically and purposively shares, blends and co-creates different types of knowledge with an emphasis on solution; and on how the knowledge generates visible and tangible products. We believe that such visible outputs demonstrate authenticity in the co-creation process (Cooke et al. 2016). Used throughout the process, participants can see their contribution, and shared decision making about what knowledge is included and taken forward can be visibly traced back from origin to end of process.

### Influence on participants

Paying attention to the process is particularly pertinent in collaborations between research and health systems partnerships [48], and in working with service and end users [9]. Boivin et al. [9] suggest diverse partnerships require consideration of credibility of each voice, legitimacy of knowledge each person brings and contributes, and paying attention to power.

Collective making addresses aspects related to power including ‘Language’, ‘Self-expression’ and ‘Presence’ [49–51]. Professional and disciplinary specific language is exclusive. Even with efforts to use ‘lay English’, there are different ways of interpreting words. Assumptions are often made that the same interpretation is taken away by different stakeholders. Words, particularly spoken words are also transient, with no sustained presence, making them easy to forget, ignore, disregard or dismiss. To quote Augusto Boal [52]:

“Words are emptinesses that fill the emptiness (vacuum) that exists between one human being and another. Words are lines that we carve in the sand, sounds that we sculpt in the air. We know the meaning of the word we pronounce, because we fill it with our desires, ideas and feelings, but we don’t know how that word is going to be heard by each listener.”

Using self-created ‘things’ to support and facilitate dialogue between people from different backgrounds enables them to use symbology, metaphors and visual representations meaningful to them. These ‘things’ create a unique language that sits outside of individual professions and disciplines and yet inside everyone’s ability; it is both uncommon to all yet common to all. It is self-expression, enabling each individual to express their view in their way. Finally, it gives everyone’s individual contribution a physical, visible, tangible presence, making it incredibly difficult for others in the group to dismiss or ignore. In situations where designers make things on behalf others (such as the R2D2 model in the Head-Up project as described ealier in Prototyping section), there is an intrinsically empowering quality for the stakeholders when they make a suggestion and it is turned into a tangible, physical ‘things’ by others. It demonstrates that what they are saying is being listened to - and acted upon.

These techniques address power differences, level hierarchies and connect with the hearts and minds of participants. The Lego Serious Play (LSP) methodology [53, 54] uses Lego bricks to build metaphorical representations of thoughts, ideas, experiences and feelings. Individuals build a model in response to a specific question and everyone is facilitated to explain their model, referring to its physical features as points to illustrate their thinking. Then, through a variety of mechanisms, individuals’ models (or elements of them) are combined, accompanied by explanations. Such approaches can contribute to collectively defining problems, developing mutual understandings, and collectively defining solutions. It can be a mechanism for explaining and sharing abstract ideas, which is especially useful when working with disparate groups of stakeholders. LSP is just one approach. Other practices might be drawing or role play and performance, but all require some time to ‘make’ individually and then to build from the individual contribution to the collective. This externalizing and metaphorical representation of different perspectives enables the group to collectively negotiate conflict through the made things, making it less personal.

With our focus on inclusion, we are also mindful of what methods of ‘making’ are used ensuring they are contextually appropriate for the target audience [55]. For example, when making things with children and young people we have effectively used the digital storyboarding technique called ‘BitStrips’ (No longer available). A storyboard is a sequence of pictures that tells a story, like a cartoon in a comic. ‘BitStrips’ was a digital resource for building storyboards with a library of ‘elements’ to construct each frame. It included characters, environments, actions, words and more. The application allowed users to create a context (office, school room, kitchen, park etc.), illustrate the weather, build avatars (people and animals), convey emotions and moods, insert tools, devices, props to use or fit into the context, speech bubbles or cartoon features. Tools like it can overcome the challenge of a ‘blank sheet of paper’, as the software walks users through the process. In this way, the concerns of ‘I can’t draw’ or ‘I’m not creative enough’ are avoided. We have found this is an accessible and ‘safe’ way for young people to tell a story to a wider group of stakeholders, yet less accessible to older people.

In summary, ‘collective making’ techniques help to level power based on language, reduce the use of jargon, enable self-expression, give tangible presence to each participant’s contribution and help to navigate conflict.

### Influence on knowledge

Making things influences the knowledge and learning in individual participants. As referred to earlier, collective making is a strategy designers use to help them to think using their cognition and their perception. In certain paridigms, the tendancy is to separate thinking from perceptions. Words (spoken or written) are the predominant tool of the mind; the ways in which cognitive processes and outcomes are expressed. Yet when engaging with practitioners and with lay people experiencing ill health, where the perceptual understanding of their experiences is as important as the cognitive reflections, this form of enquiry with them becomes a powerful way of enabling people to think, reflect and communicate their experiences fully. Making is inherently a reflective [28] and absorbing process [56] that can unlock memories and embody both explicit and tacit knowledge. The act of making gives the individuals engaged in the process the space for their unconscious mind to dwell on the whys and wherefores of what is being made – allowing unconscious thoughts to surface and be shared with others, therefore influencing the collective making process.

Our contention is that the making process itself influences both access to, and utility of, different types of knowledge in the co-creation process, in that it helps to make tacit knowledge more explicit. Tacit knowledge can be defined as skills, ideas or ‘know how’, as well as beliefs and mental models that enable this [57]. Often the tacit knowledge holder is unaware of this knowledge, and does not understand how it may be valuable to others [58]. For this reason, tacit knowledge is difficult to share and make explicit [57]. Research in new product development has highlighted that personal contact, networking and use of metaphor can help to communicate and share tacit knowledge with others [58]. Additionally, Collins [59] has identified a number of subgroups of tacit knowledge that we think are particularly pertinent for discovery through co-design approaches. These include ‘concealed’, ‘ostensive’ and ‘uncognized’ tacit knowledge. ‘Concealed’ tacit knowledge includes skills and techniques learned through practice; the ‘tricks of the trade’. Ask someone to describe how they use a device or tool and they might miss out a few steps that they never miss in practice. Ask them to show you and you will get the complete picture. Ask them to build a model and show you and you will find that both user and observer learn. ‘Ostensive’ tacit knowledge is where words may not be available to convey knowledge where gestures can. Here performance or photos may help to make tacit knowledge more explicit. And finally, ‘uncognized’ knowledge is where a successful experimenter may be unaware of factors that contribute to their problem solving, whilst others who watch can. We would suggest that making things helps to capture and make explicit these types of tacit knowledge.

We have explained earlier the process of building from the individual to the collective during the making process, and that a series of prototypes provides a visible trail of joint learning. These can explain at each stage what was learned and how it contributed to the next phase, and why certain decisions were taken as a group or avenues pursued, so what ends up on the ‘cutting room floor’ is still useful. This access and utilisation of tacit knowledge will enable a much wider systems perspective. Equally as relevant here is that the act of collective making can also transform more formal codified knowledge into forms that can be accessed and synthesised by the whole group. Complex research findings can be transformed to more embodied forms through roleplay or narrative descriptions.

### Influencing knowledge implementation

Collective making produces outputs that act as ‘boundary objects’. A boundary object is defined as ‘a construct that has potential to improve the uptake transfer and innovation of research findings, technology and other intellectual property across the fields of social policy, organisation and management and commercial and public services’(p70) [60]. Because collective making produces things that embody the joint knowledge created, mindful of context of its application, we suggest they are more likely to be actionable [4].

For example, the neck brace developed through Head up is now being used by people with MND and is a product on the market. It embodies testimony of people with MND, professionals caring for them and MND specialist researchers and therefore fulfils the definition of a boundary object. The practitioners who prescribe it know how it can impact on the quality of life for people with MND. It also encourages ownership of the product, because it has been co-created with the end users. This was highly visible in the Head-Up project where stakeholders in the co-creation process have continued investing significant time and effort in the project to actively champion and support its wider adoption.

A further influence on implementation is that the practical process of iteratively making things together enabled the group to unconsciously and consciously consider practical implications of using something in a given context and for a given set of users that might otherwise be harder to consider.

### How does this fit into the existing KMb landscape?

In the field of co-production, co-design and Mode 2 research, it would be remiss not to mention the Integrated Knowledge Translation (iKT) approach [61, 62]. In the words of Graham et al. “…this category of KT is similar to participatory research or Gibbons’ Mode 2 knowledge production…”. Depending on perspective, one could argue that Collective Making might be interpreted as a specific (but novel) form of iKT – or perhaps iKT is a specific interpretation of co-production, and Collective Making is a specific form of co-production. Kothari and Wathen refer to ‘visualisations techniques’ as a possible tool for engagement within iKT [62]. But there is limited consideration of how the process of *making* such visualisations might in itself have any value for knowledge creation and sharing.

We see our model as functioning within the principles described by Greenhalgh to co-create enhanced forms of evidence that blend research knowledge with experiential (patient and professional) and contextual knowledge, creating more implementable knowledge. The model could, theoretically, be used at any stage of the process, either starting from defining a research question or a priority based on research evidence, patient and professional experience and contextual factors. Or carrying out research, where technical research work might be undertaken by researchers but definition of research questions, methods, data collection, analysis and evaluation might all be collaboratively undertaken using this model. Or it could be used to take an evidence based policy or guideline and work locally with researchers, patients and professionals to determine how it might best be implemented and made to fit their context, lives and local demographic.

We believe our approach is not one that necessarily sits in isolation from other KMb or implementation strategies, and it could be complimentary to many that already exist. Its uniqueness, relative to all other models of co-production, iKT, KMb or implementation, lies in its mechanism (collective making) for engaging diverse people in a collaborative process and the impact this mechanism has on communication, redistributing power and eliciting and sharing different forms of knowledge. To our knowledge, the specific notion of using ‘Collective making’ to do co-production or iKT has not be previously defined or mentioned.

## Conclusions

The paper proposes that ‘collective making’ within a creative process of co-design, provides techniques and opportunities for Mode 2 learning that will facilitate KMb between stakeholders. It should be considered along with other techniques as a resource to the KMb community.

Design is essentially a practical and pragmatic discipline that combines knowledge creation *and* knowledge use. Engaging with end users on wicked problems to make useful products and find solutions is core to design practice. Co-creation is not easy, as such, we concur with Greenhalgh and colleagues in so much that a lack of attention to the principles of successful co-creation will result in failure. Collective making in co-design satisfys the principles and also addresses many of the broader challenges of coproduction so has many characteristics of a possible candidate to operationalise mode 2 KMb.

Co-design and designers can provide expertise and methods to develop dynamic and adaptive community-academic partnerships. During this paper, we have outlined how ‘collective making’ adopts a systems approach, it unpacks and explores human experience as its driving force, and it is a creative enterprise that develops actionable outputs as boundary objects. We have described how many of the techniques are empowering and pay attention to voice of each participant, address power sharing, and adopt an egalitarian approach. Additionally, we have suggested that collective making might have a unique influence on the participants, on the knowledge uncovered and created, and on the products developed, and their potential for implementation. The made things or prototypes are a physical embodiment of co-created and blurred knowledges. Importantly, some of the techniques uncover and use participants’ tacit knowledge of participants. Finally, we suggest that because the process ensures collective ownership of such outputs and makes them visible, it demonstrates the authenticity of the co-creation process.

The next phase of development for the model is to start to test the emergent theory empirically. Process evaluation of case studies and subsequent testing and development of the model will help to establish its place in the panoply of KMb approaches. The authors feel that collective making could sit in the ‘process models’section of Nilsen’s categories of theorys, models and frameworks of implementation science [63] and support the continued development and recognition of mode 2 KMb as not only a scientifically and theoretically valid approach, but one that practically delivers benefit to the health and wealth of society.

In terms of implications for practice, the use of collective making may be novel in the world of health care research, but as we have outlined in the background, aspects of collective making are taught in design courses across the globe. We would argue that as for many of today’s challenges, trans-disciplinary approaches are needed that will blend the skills, knowledge and experience of trained designers with those of the growing community of knowledge mobilisers, researchers and other key stakeholders in Health and social care.

## Abbreviations

iKT: Integrated Knowledge Translation
KMb: Knowledge Mobilisation
